# Impact of electron beam irradiation on the chlorophyll degradation and antioxidant capacity of mango fruit

**DOI:** 10.1186/s13765-021-00592-8

**Published:** 2021-02-03

**Authors:** Truc Trung Nguyen, Apiradee Uthairatanakij, Varit Srilaong, Natta Laohakunjit, Masaya Kato, Pongphen Jitareerat

**Affiliations:** 1 Division of Postharvest Technology, School of Bioresources and Technology, King Mongkut’s University of Technology Thonburi, Bangkok, 10140 Thailand; 2 Postharvest Technology Innovation Center, Commission of Higher Education, Bangkok, 10400 Thailand; 3 Division of Biochemical Technology, School of Bioresources and Technology, King Mongkut’s University of Technology Thonburi, Bangkok, 10140 Thailand; 4 Department of Bioresource Science, Faculty of Agriculture, Shizuoka University, Shizuoka, 422-8529 Japan

**Keywords:** Ionizing irradiation, Chlorophyll degradation, Reactive oxygen species, Antioxidant capacity

## Abstract

At the present, the mechanism of chlorophyll degradation in response to ionizing irradiation in harvested fruits have not been examined. To understand the effect of electron beam (E-beam) irradiation on the chlorophyll degrading pathway in relation to chlorophyll degrading enzymes activity, reactive oxygen species (ROS) and antioxidant capacities of harvested mangoes stored at 13 °C for 16 days were studied. E-beam-treated fruit significantly suppressed the activities of chlorophyll degrading enzymes especially pheophytinase (PPH) and chlorophyll degrading peroxidase (Chl-POX) in the late stage of storage. This resulted in the chlorophyll content being maintained. However, E-beam irradiation did not affect the activities of chlorophyllase (Chlase) and magnesium de-chelatase (MD). The respiration rate, ethylene production, ROS accumulation (hydrogen peroxide [H_2_O_2_] and superoxide radical [O^−**.**^_2_]) immediately increased after E-beam treatment, following which they significantly decreased in comparison to the control. E-beam treatment enhanced the fruit’s antioxidant capacity by activating the activities of catalase (CAT) and ascorbate peroxidase (APX) and glutathione (GSH) content, and inactivated the activity of superoxide dismutase (SOD). Further, it did not affect the activity of glutathione reductase (GR) and glutathione disulfide (GSSG), vitamin C content, or total phenolic content. These results imply that E-beam treatment has the potential to delay chlorophyll degradation by suppressing the Chl-POX and PPH activities as well as reduce ROS production via CAT, APX, and SOD activities and GSH content.

## Introduction

Mango (*Mangifera indica* L.) is a popular tropical fruit for its dietary fiber, vitamin C, and pigments and is an excellent source of antioxidants and phytochemicals [1–3]. After harvesting, it ripens within 4 days under ambient temperature [4]. One of the most visible changes during the postharvest ripening of mangoes is chlorophyll degradation [5], which further leads to the yellowing of its skin quickly. The yellow appearance indicates that mango fruit cannot be stored long periods because it is consequent senescence in shortly. Therefore, the delaying of the chlorophyll degradation is one criteria that help to extend the storage life of mango which it can be exported for long distance markets.

Chlorophyll is a naturally green pigment biosynthesized in higher plants. Chlorophyll is a photochemically active compound that also plays a role in human health [6]. During storage, chlorophyll degradation takes place in two stages: in the early stage, dephytylation and magnesium dechelation take place. Chlase is one of first enzymes in the chlorophyll degradation process and removes the phytol group in chlorophyll a structure to obtain chlorophyllide a [7, 8]. Following this, chlorophyllide a releases magnesium ion to form pheophorbide a, which is catalyzed by MD [9, 10]. Following this, the late stage consists of the oxygenolytic cleavage of pheophorbide a, reduction of the red chlorophyll catabolite, and modification of the primary fluorescent chlorophyll catabolite. Pheophorbide a oxygenase (PAO) converts pheophorbide a to red chlorophyll catabolites, which is followed by a reduction in the red chlorophyll catabolite induced by red chlorophyll catabolite reductase (RCCR), resulting in a colorless fluorescent product known as the primary fluorescent chlorophyll catabolite. Then, the primary fluorescent chlorophyll catabolite is transformed to fluorescent chlorophyll catabolites via a demethylation and hydroxylation process [11, 12]. Additionally, previous researchers have reported that Chl-POX can convert chlorophyll a to 13^2^-hydroxychlorohyll a, which is a fluorescent chlorophyll catabolite, in the presence of H_2_O_2_ and phenolic compounds such as *p*-coumaric acid, apigenin, and 2,4-dichlorophenol [13, 14]. Further, chlorophyll is degraded by the oxidation of its phytyl chain, caused by reactive oxygen species (ROS) or oxy free radicals in higher plants such as *Petroselinum sativum* [15] and *Posidonia oceanica* [16].

Ionizing irradiation is a form of non-thermal technology that is widely used to solve various agricultural problems, such as inactivating the food-borne pathogens, suppressing the sprouting of tuber crops, delaying the ripening of harvested produce, and controlling postharvest losses caused by insect and fungal infestations [17]. Ionizing gamma irradiation has been reported to maintain chlorophyll content in quince fruit [18], tomatoes [19], plums [20], pears [21], fenugreek, and spinach [22] whereas ionizing E-beam treatment could reduce postharvest disease, maintain firmness and also delay the color change of mango fruit [23]. However, there is a lack of knowledge about the effect of ionizing E-beam irradiation on chlorophyll-degrading enzymes and the correlation between chlorophyll degradation and ROS production in mangoes. Therefore, the present study is the first report to show that ionizing E-beam irradiation delays chlorophyll degradation by suppressing chlorophyll-degrading enzyme activities and ROS production as well as enhances antioxidant capacity in harvested mangoes.

## Materials and methods

### Mango samples and electron beam treatment

Mature green mangoes cv. Nam Dok Mai Si Thong were harvested from a farm in the Ratchaburi province (90–100 days after fruit set) and transported to a research laboratory at the Division of Postharvest Technology within 2 h. The fruits were checked for uniformity in size, weight (350–370 g), color, and shape, and they were free of any visible defects and infestations. They were surface disinfested with a solution of 0.1 g L^−1^ sodium hypochlorite and airdried for 2 h before treatment. Our preliminary test showed that E-beam treatments of > 1.0 kGy causes physical injury to the mango peel after 24 h of treatment, whereas 0.5 kGy causes no injury. Thus, 0.5 kGy E-beam irradiation was chosen for this experiment.

One hundred twenty fruits were placed in a corrugated paper box (40 × 20 × 10 cm; 15 fruits per box) and treated with 0.5 kGy E-beam at an ambient temperature of 28 ± 2 °C. The irradiation was carried out at the Thailand Institute of Nuclear Technology (TINT), Nakhon Nayok province. An E-beam linear accelerator (AECL accelerators, Kanata On, Canada) of 10 meV with a pulse repetition frequency (PRF) of 60 Hz was used, and the under beam conveyor (UBC) speed was controlled at 0.024 m s^−1^ to treat the mangoes. Eight alanine dosimeters (TSS Quotation-SP, Thai Sterilization Services Co. Ltd.) per box were attached to the top and bottom of each fruit’s surface to measure the desired dose. The dosimeters were then read using an alanine dosimeter reader (Electron spin resonance spectroscopy) (Additional file 1). None of the fruits exposed to the E-beam (120 fruits) were used as the control.

The dosage uniformity value of the treated fruits (D_max_/D_min_) was satisfactory at 1.81; the accepted range for the uniformity ratio value of an electron beam is required to be between 1.5 and 2 or even higher [24]. After irradiation, all the fruit samples were stored at 13 °C for 16 days. The samples were then randomly collected to evaluate the chlorophyll content, activity of chlorophyll-degrading enzymes, ROS, antioxidant capacity, respiration rate, and ethylene production during the initial days and then after 4-day intervals.

### Determination of the peel color

The color changes in the mango peel were measured using a colorimeter (Model CR-400, Konica Minolta, Japan) every 4 days. The colors were determined for three positions of the fruit’s surface (upper, middle, and lower parts) and averaged to obtain one value per fruit. The values for L*, a*, b*, and hue angle were recorded. The total difference in the color value (ΔE is the color change between two measurement times) was calculated using the following formula:

$$\Delta E = \sqrt {\left( {L_{0}^{*} - L_{t}^{*} } \right)^{2} + \left( {a_{0}^{*} - a_{t}^{*} } \right)^{2} + \left( {b_{0}^{*} - b_{t}^{*} } \right)^{2} }$$

where $L_{0}^{*}$, $a_{0}^{*}$, and $b_{0}^{*}$ indicate the color values of the samples on the initial day (day 0), and $L_{t}^{*}$, $a_{t}^{*}$, and $b_{t}^{*}$ indicate the values of the samples every 4 days throughout the storage period.

### Determination of chlorophyll content

Chlorophyll was extracted with *N*,*N*-dimethylformamide at 4 °C in the dark for 24 h. The solution was filtered through Whatman No.1 filter paper. The absorption value of the chlorophyll content was measured at 664 and 647 nm using a spectrophotometer (UV-1800; Shimadzu Co., Kyoto, Japan) and expressed as mg kg^−1^ of fresh weight [25].

### Substrate preparation of chlorophyll degrading enzymes

#### Acetone powder

A 5 g sample of mango peel was homogenized in 100 mL cold acetone (− 20 °C). The homogenate was filtered through filter paper (Whatman No. 1). The residue was washed with cold acetone and ethyl acetate to elute pigments and then completely dried under a vacuum pump at room temperature (25 ± 2 °C) for 20 min. It was then transferred to a desiccator jar containing silica gel for 1 day and stored at − 20 °C [26].

#### Chlorophyll a

Chlorophyll a was prepared from spinach leaves by following method of Aiamla-Or et al. [27]. Spinach leaves (5 g) were homogenized for 3 min in 20 mL cold acetone (− 20 °C). The homogenate was filtered through a filter paper (Whatman No. 1), and the filtrate was treated with 1,4-dioxane and distilled water in the ratio 4:2:3 (v/v), followed by incubation at 4 °C in the dark for 1 h. The filtrate was centrifuged at 10,000×*g* for 15 min at 4 °C, after which the pellets were dissolved again in the mixture of acetone, 1,4-dioxane, and distilled water (15:2:5 v/v) and kept at 4 °C in the dark for 1 h. Subsequently, the soluble pellets were centrifuged at 10,000×*g* for 15 min at 4 °C. Afterward, the pellets were dissolved in petroleum ether and stored at − 20 °C until the individual pigments were separated using sugar powder column chromatography. Finally, 0.5 g L^−1^ of chlorophyll a was prepared in acetone.

#### Chlorophyllin a

The solution of chlorophyll a in acetone (0.5 g L^−1^) was deposited into petroleum ether. The chlorophyll a in the petroleum ether phase was washed three times with distilled water to obtain concentrated chlorophyll a. Afterward, chlorophyllin a was precipitated with 30% potassium hydroxide (KOH) in methanol. Further, the solution was centrifuged at 16,000×*g* for 15 min at 4 °C and dissolved in distilled water. Subsequently, chlorophyllin a was adjusted to pH 9.0 using 1 M Tricine [28].

#### Pheophytin a

Pheophytin a was prepared from chlorophyll a in acetone (0.5 g L^−1^) by adding one drop of 0.1 N hydrochloric acid (HCl). After treatment for 2 min, the pheophytin a solution was neutralized by 0.1 N sodium hydroxide (NaOH) until a pH of 7.0 was reached [29].

### Chlorophyll degrading enzyme activity assay

An extraction of crude enzymes was conducted using 0.5 g of mango peel acetone powder mixed with 15 mL of 10 mM phosphate buffer (pH 7.0), containing 0.6% CHAPS (3-[(3-cholamidopropyl) dimethylammonio]-1-propanesulfonate) for Chlase. For Chl-POX, a mango peel acetone powder (0.5 g) was suspended in 15 mL of 50 mM phosphate buffer (pH 7.0). In the case of PPH, a 50 mM Tris–HCl (Tris (hydroxymethyl) aminomethane-hydrochloric acid) buffer (pH 8.0) was used instead of 50 mM phosphate buffer (pH 7.0). For MD, a mango peel acetone powder (0.5 g) was assorted with 15 mL of 10 mM phosphate buffer (pH 7.0) containing 50 mM potassium chloride (KCl) and 0.24% Triton-X 100. The mixture solutions were stirred at 4 °C for 1 h and filtered with two layers of Miracloth. The filtrate was then centrifuged at 16,000×*g* at 4 °C for 15 min. The supernatant was used as the crude enzyme extract. The protein content of the crude extract was determined following Bradford’s method [30].

#### Chlase activity

Chlase activity was analyzed following the Aiamla-Or et al.’s method [26]. The reaction mixture contained 0.5 mL of 0.1 mM phosphate buffer (pH 7.5), 0.2 mL of 0.5 g L^−1^ chlorophyll a in acetone solution, and 0.5 mL of crude enzyme solution. The reaction mixture was incubated at room temperature (25 ± 2 °C) for 60 min, and the enzyme reaction was inhibited by adding 4 mL of acetone. Chlorophyllide a was separated by adding 4 mL of hexane. The upper phase contained the remaining chlorophyll a, while the lower phase contained chlorophyllide a. The activity was spectrophotometrically detected by the formation of chlorophyllide a at 667 nm (76.79 mM^−1^ cm^−1^) per min per mg of protein.

#### Chl-POX activity

Chl-POX activity was measured in accordance with Yamauchi et al. [31] but with some modifications. The reaction mixture contained 0.5 mL of crude enzyme extract, 0.1 mL of 1.0% Triton-X 100, 0.1 mL of 5 mM *p*-coumaric acid, 0.2 mL of 0.5 g L^−1^ chlorophyll a in acetone solution, 1.5 mL of 0.1 mM phosphate buffer (pH 5.5), and 0.1 mL of 0.3% hydrogen peroxide. The reaction mixture was incubated at 25 ± 2 °C for 40 min. Enzyme activity was spectrophotometrically determined by measuring the decrease in chlorophyll a at 668 nm per min per mg of protein at 25 ± 2 °C.

#### PPH activity

PPH activity was determined by using the method given by Kaewsuksaeng et al. [29]. The reaction mixture contained 0.5 mL of 50 mM Tris–HCl buffer (pH 8.0), 0.25 mL of pheophytin a (0.043 g L^−1^) in acetone solution, and 0.25 mL of crude enzyme extract. The mixture was incubated at 25 ± 2 °C for 40 min and then inhibited by adding 2 mL of acetone. The PPH activity was spectrophotometrically detected based on the pheophorbide a formation at 665 nm (44 mM^−1^ cm^−1^) per min per mg of protein.

#### MD activity

MD activity was spectrophotometrically determined using chlorophyllin a by measuring the absorbance of pheophorbin a formation at 686 nm [32]. The reaction mixture, which contained 0.5 mL of 50 mM Tris-tricine buffer (pH 8.8), 0.1 mL of chlorophyllin a (OD_687_ = 0.4), and 0.2 mL of crude enzyme extract, was incubated at 37 °C for 3 min. MD activity was expressed as the increment of OD at 686 nm per min per mg of protein under the test conditions.

### Ethylene production and respiration rate

One fruit was incubated in plastic box (1.4 L in volume) for 3 h at 13 °C. One milliliter of gas sample from the headspace of the plastic box was drawn using a syringe and injected into a gas chromatograph (Shimadzu GC-14B, Bara scientific, Japan) with a thermo-conductivity detector for carbon dioxide analysis and a flame ionization detector for ethylene analysis. Each treatment consisted four replicates (boxes).

### Hydrogen peroxide and superoxide radical

The hydrogen peroxide (H_2_O_2_) content was measured using Wu et al.’s method [33], albeit with some modifications. Peel fruit tissue (0.5 g) was mixed with 8 mL of 5% cold trichloroacetic acid (w/v) and was subsequently homogenized and centrifuged at 10,000×*g* at 4 °C for 10 min. The reaction mixture was prepared using 0.5 mL of the supernatant, 4 mL of 5% trichloroacetic, and 0.5 mL of assay reagent containing 500 µM ferrous ammonium sulfate, 50 mM sulfuric acid (H_2_SO_4_), 200 µM xylenol orange, and 200 mM sorbitol. The reaction mixture was incubated at 25 ± 2 °C for 45 min. During the incubation period, the hydrogen peroxide molecule oxidized Fe^2+^ to Fe^3+^ ion, which was determined by measuring the absorbance of the ferric-xylenol orange complex at 560 nm. The absorbance values were calibrated to a standard curve, generated using the known concentrations of H_2_O_2_.

The production rate of superoxide radical (O^−**.**^_2_) was analyzed using the method given by Elstner and Heupel [34]. Peel tissue samples (1 g) were homogenized with 8 mL of 65 mM phosphate buffer (pH 7.8) containing 1 mM ethylenediaminetetraacetic acid (EDTA), 1% polyvinylpolypyrrolidone (PVPP w/v), and 0.3% Triton X-100. The extraction mixture was centrifuged at 5000×*g* for 15 min at 4 °C. After centrifugation, 0.5 mL of the supernatant was mixed with 1 mL of 50 mM phosphate buffer (pH 7.8) and 0.5 mL of 10 mM hydroxylamine hydrochloride and subsequently incubated at 25 ± 2 °C for 20 min in dark conditions. A 1 mL sample of the above reaction mixture was added to 1 mL of 19 mM *p*-aminobenzene sulfonic acid and 1 mL of 7 mM α-naphthylamine to formulate a new mixture, which was then incubated at 25 ± 2 °C for 20 min in dark conditions. The O^−**.**^_2_ content was calculated based on a comparison of absorbance with a standard curve (using sodium nitrite as the standard) at 530 nm. The O^−**.**^_2_ production rate was expressed as µmol NO_2_ kg^−1^ s^−1^ of fresh weight.

### Antioxidant compounds and enzymatic antioxidant activities

#### GSH and GSSG assay

Crude extracts for the analysis of total glutathione were prepared by homogenizing 2 g of mango peel in 10 mL of cold 0.9% NaCl (w/v) containing 5 mM EDTA, which was then centrifuged at 17,000×*g* at 4 °C for 10 min. For GSH, 5 mL of the above crude extract was precipitated with 5 mL of cold 30% trichloroacetic acid (w/v) for 5 min. Further, it was centrifuged at 17,000×*g* at 4 °C for 10 min to remove protein [35]. The total and GSH contents were measured using the method stated by Griffth [36] but with some modifications. The reaction to determine the total glutathione content was facilitated by mixing 1.4 mL of 0.3 mM nicotinamide adenine dinucleotide phosphate (NADPH) and 200 µL of 6 mM DTNB (Ellman’s reagent; 5,5′-dithiobis-(2-nitrobenzoic acid)) in 100 mM phosphate buffer with 5 mM EDTA disodium salt (pH 7.5), 0.02 mL of glutathione reductase (50 U mg^−1^ protein from yeast), and 0.4 mL of crude extract. The GSH content was measured using 0.2 mL of 6 mM DTNB, 1.4 mL of 100 mM phosphate buffer with 5 mM EDTA disodium salt (pH 7.5), and 0.4 mL of crude extract. The solution of total glutathione and GSH assay was incubated at 25 ± 2 °C for 1 h and subsequently measured using a spectrophotometer at 412 nm. The GSSG content was calculated using total glutathione subtract to the content of GSH in a similar sample. The GSH and GSSG contents were calculated using a standard graph and expressed in terms of milligrams of glutathione equivalents per kilogram of fresh weight.

#### Total phenolic content

The crude extract was prepared following the method given by Ribeiro et al. [37] but with some modifications. 2.5 g of mango peel was mixed with 10 mL of methanol:water (60:40 v/v). The sample was homogenized and then centrifuged at 15,000×*g* for 20 min at 4 °C. The supernatant was used to determine the total phenolic content using the Folin–Ciocalteu method, as described by Singleton et al. [38]. For the reaction mixture, aliquot of 0.05 mL of the extract was added to 0.25 mL of Folin–Ciocalteu reagent, followed by an addition of 0.75 mL of 7.5% sodium carbonate solution and 2 mL of distilled water. The mixture was vortexed and incubated in a water bath at 40 °C for 30 min. The absorbance was measured at a wavelength of 750 nm using a spectrophotometer (UV 1800, Shimadzu, Kyoto, Japan). A blank sample consisting of distilled water and the Folin–Ciocalteu reagent was used for comparison purposes. The results were expressed in terms of grams of gallic acid equivalents (GAE) per kilogram of fresh weight.

#### Vitamin C content

Vitamin C content was measured following Roe et al.’s method [39]. Five grams of mango peel were homogenized with 20 mL of cold 5% metaphosphoric acid and then filtered through Whatman No. 1 filter paper. Aliquot of the filtrate was centrifuged at 17,000×*g* for 20 min at 4 °C. The reaction mixture was prepared using 0.4 mL of filtrated solution and 0.2 mL of 0.02% di-indophenol. This was added to 0.4 mL of 2% thiourea and 0.2 mL of 2% dinitrophenol hydrazine and then incubated at 50 °C for 1 h. After incubation, 1 mL of 85% sulfuric acid was added, and the reaction mixture was incubated at an ambient temperature for 30 min. The absorbacne of vitamin C was measured at 540 nm using a spectrophotometer (UV-1800; Shimadzu Co., Kyoto, Japan) and expressed as g kg^−1^ of fresh weight.

#### SOD activity

The crude enzymes were extracted using 2 g of peel tissue in 10 mL of 65 mM phosphate buffer (pH 7.8) containing 1% polyvinyl pyrrolidine and 1 mM EDTA. They were then homogenized and centrifuged at 15,000×*g* at 4 °C for 20 min. The enzyme activity was determined according to the indirect spectrophotometric method given by Elstner and Heupel [34]. The reaction mixture contained 0.5 mL of 65 mM phosphate buffer (pH 7.8) and 1 mL of xanthine oxidase from bovine milk containing 150 μg protein, 0.1 mL of 1.5 μmol xanthine, 0.1 mL of 1 μmol hydroxylamine hydrochloride, and 0.3 mL of crude enzyme. Following this, the mixture was incubated at 25 ± 2 °C for 20 min in the dark. 0.5 mL of the above reaction mixture was removed and added to 0.5 mL of 19 mM *p*-aminobenzene sulfonic acid and 0.5 mL of 7 mM α-naphthylamine to obtain a new mixture, which was then incubated at 25 ± 2 °C for 20 min in the dark. SOD activity was determined by measuring the absorbance of the reaction mixture at a wavelength of 530 nm. One unit of SOD was defined as the enzyme amount that inhibited the nitrite dioxide formation rate by 50% per min per mg of protein.

#### CAT and APX activities

The crude enzymes were extracted adding 2 g of peel tissue to 10 mL of 100 mM phosphate buffer (pH 7.5) containing 1% polyvinyl pyrrolidine and 1 mM EDTA, which was subsequently homogenized and centrifuged at 15,000×*g* at 4 °C for 20 min. The supernatant was used to determine the CAT and APX activity [40].

CAT activity was determined based on the decreasing concentration of H_2_O_2_ (extinction coefficient 39.4 M^−1^ cm^−1^) at a wavelength of 240 nm for 90 s. The reaction mixture contained 0.02 mL of 30% H_2_O_2_, 0.78 mL of 100 mM phosphate buffer added to 1 mM EDTA (pH 7.5), and 0.2 mL of crude extract enzyme.

APX activity was measured based on the oxidation of ascorbate by H_2_O_2_ (extinction coefficient 2800 M^−1^ cm^−1^) at a wavelength of 290 nm for 90 s. The reaction mixture contained 0.05 mL of enzyme extract, 0.05 mL of 10 mM ascorbate, and 0.89 mL of 0.1 M phosphate buffer, to which 1 mM EDTA (pH 7.5) was added. The reaction was initiated by adding 0.01 mL of 20 mM H_2_O_2_.

#### GR activity

A 2 g sample of peel tissue was homogenized with 10 mL of 50 mM phosphate buffer (pH 7.0) containing 0.1 mM EDTA. The homogenate was centrifuged at 17,000×*g* for 10 min at 4 °C. The supernatant was collected and used for the enzyme assays. The GR activity was determined based on the oxidation of NADPH (extinction coefficient 6200 M^−1^ cm^−1^) at 340 nm for 1 min, as described by Rao et al. [41]. The reaction mixture (2 mL) was obtained by mixing 1.6 mL of 100 mM phosphate buffer (pH 7.8) containing 2 mM EDTA, 0.2 mM NADPH, 0.5 mM GSSG, and 0.4 mL of the enzyme supernatant.

### Statistical analysis

The obtained data were analyzed using the general linear model procedure with the statistical analysis software (SAS), version 9.0 (SAS Institute, Cary, N.C., USA), for completely randomized design experiments. The means were compared using an independent samples t-test. The value of P < 0.05, P < 0.01, and P < 0.001 expressed the statistical significance. Each treatment consisted 3 replications and each replication consisted 6 fruits. Color measurement, the color values from 6 fruits were averaged and presented as the mean of one replication. Biochemical analysis, the equal amounts of tissue samples from 6 fruits were collected, mixed well and used as one replication to assay the chlorophyll contents, chlorophyll-degrading enzyme activities, reactive oxygen species, antioxidant compounds, and enzymatic antioxidant activities. Four replications were used to assay the respiration rate and ethylene production for each treatment. The data were expressed in the form of mean ± standard error.

## Results

### Effect of E-beam irradiation on chlorophyll content and chlorophyll-degrading enzymes

**Fig. 1:**
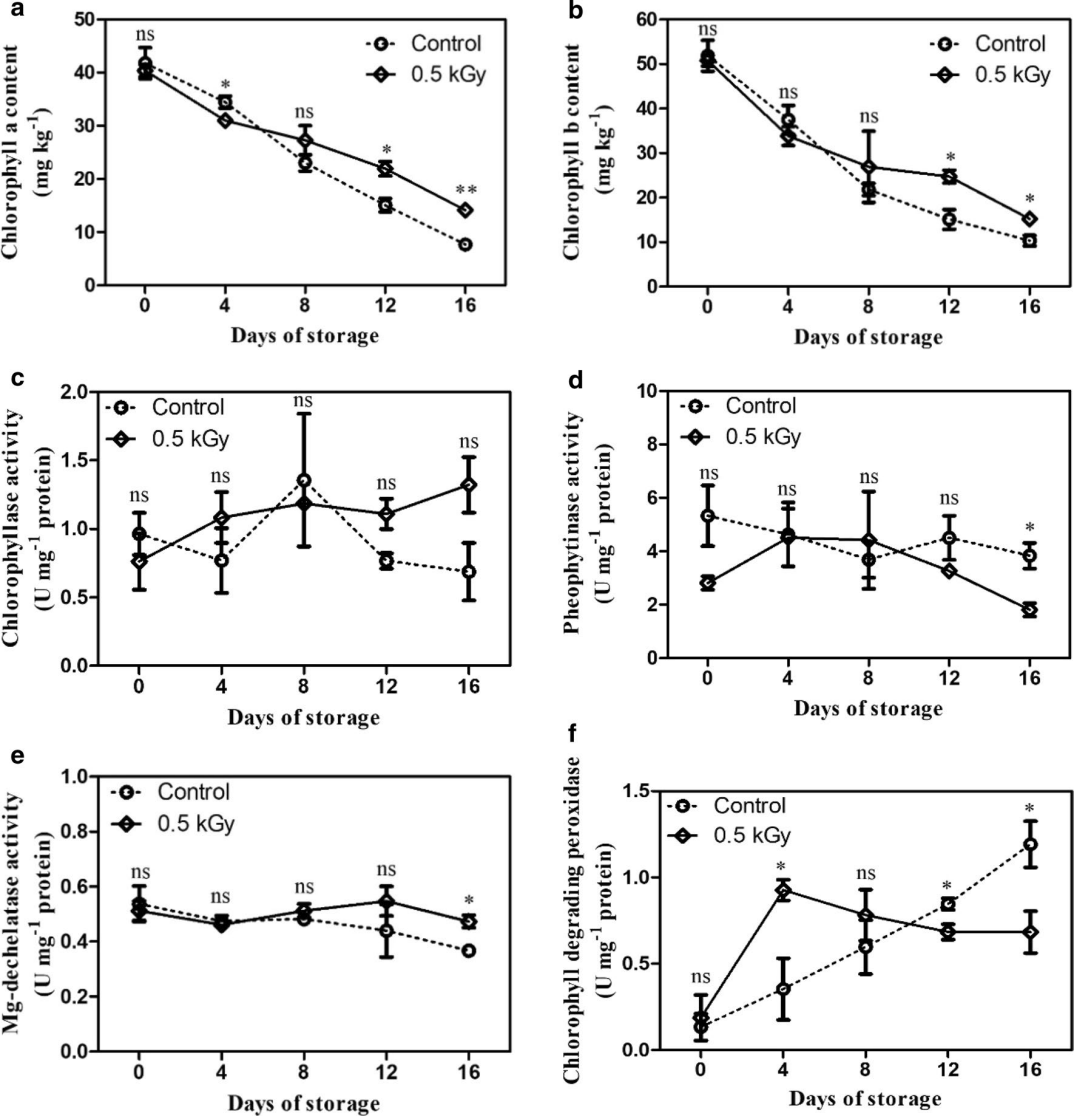
Chlorophyll a (**a**) and chlorophyll b (**b**) contents and chlorophyll degrading enzyme activities: chlorophyllase (**c**), pheophytinase (**d**), Mg-dechelatase (**e**) and chlorophyll degrading peroxidase (**f**) of mangoes after being treated with E-beam at a dose of 0 (control) and 0.5 kGy, and then stored at 13 °C for 16 days. The data are expressed as mean ± standard error. Asterisks (*) indicate significant differences between the two treatments during storage (t-test; *P < 0.05, **P < 0.01, *ns* not significant)

**Fig. 2:**
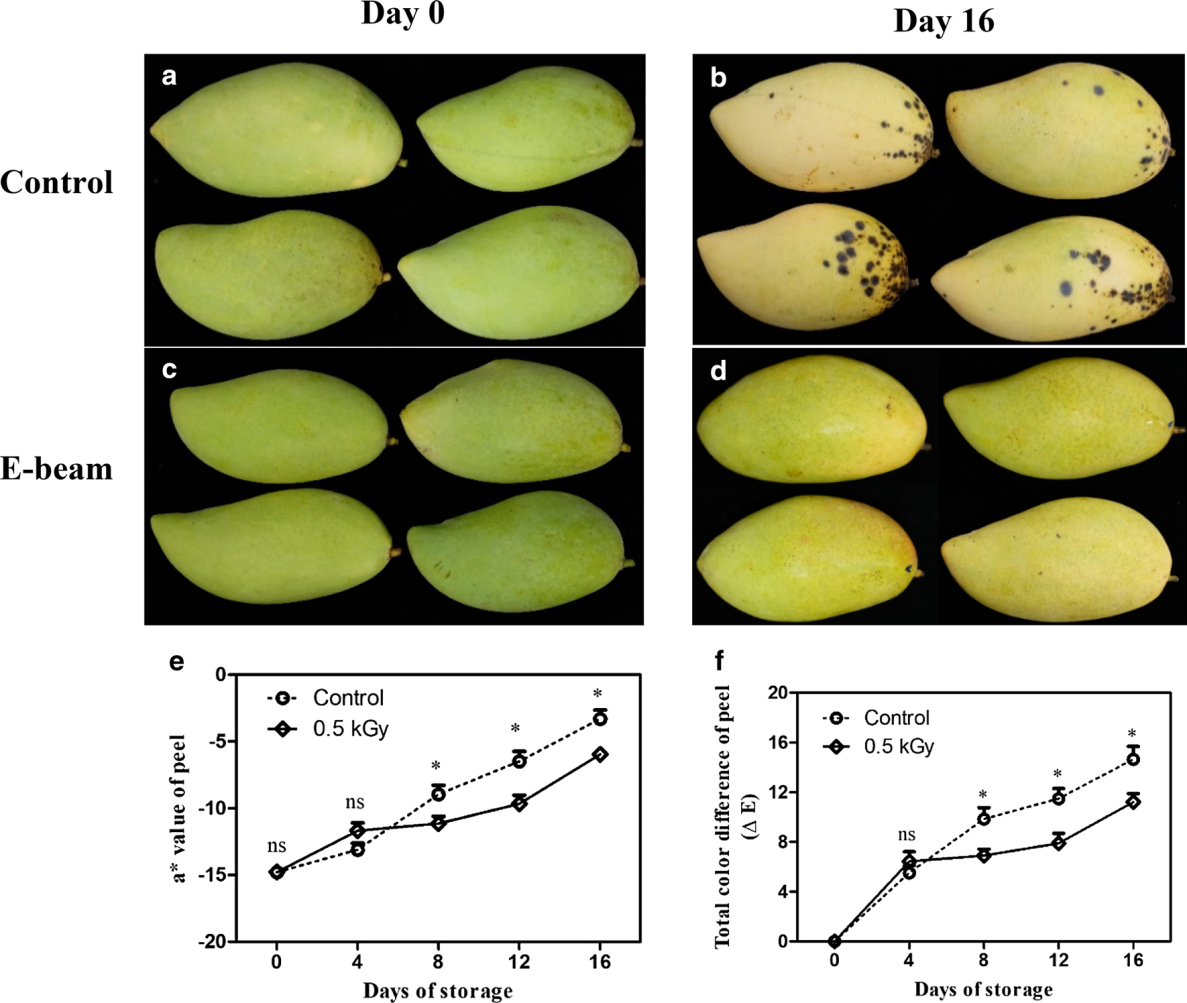
The appearance of mangoes (**a**–**d**), a* value (**e**), and total color difference (**f**) after treatment with E-beam at a dose of 0 (control) and 0.5 kGy, and then stored at 13 °C for 16 days. The data are expressed as mean ± standard error. Asterisks (*) indicate significant differences between the two treatments during storage (t-test; *P < 0.05, **P < 0.01, *ns* not significant)

Chlorophyll is the green pigment that plays a role as an indicator of fresh and healthy fruit. Chlorophyll a and b contents tended to reduce in E-beam-treated fruit and non-treated fruit throughout the storage period. On day 0, there was no significant difference in chlorophyll a and b contents of the mango peels in both the treatments. On day 4 of storage, the E-beam treatment stimulated chlorophyll degradation; afterwards the degradation of chlorophyll contents in the E-beam-treated fruit was lower than that of the non-treated fruit from day 8 until the end of the storage period (Fig. 1a, b). This result correlated with the change in color of the peel. A statistical analysis demonstrated that the E-beam-treated fruit had significantly lower values of a* (greenness) and ΔE (total difference in color) than the control fruit from days 8 to 16 (Fig. 2a–f). Thus, E-beam treatment could maintain the green color of the mango peel.

No significant difference in Chlase activity was observed between the control and E-beam-treated fruit throughout the period of storage, and their activity levels were 0.76–1.32 U mg^−1^ protein (Fig. 1c). The PPH activities of the treated and non-treated fruit were not significantly different from days 0 to 12, with a range of 2.81–5.33 U mg^−1^ protein. However, on the last day (day 16), the PPH activity of the E-beam-treated fruit was 2.12-fold lower than that of the control fruit (Fig. 1d), which is a considerable difference. The Chl-POX activity of the control fruit increased from 0.13 U mg^−1^ protein on day 0 to 1.19 U mg^−1^ protein on day 16, whereas the Chl-POX activity of the E-beam-treated fruit rapidly increased and peaked on day 4 (0.92 U mg^−1^ protein) before declining slightly until the end of storage (0.68 U mg^−1^ protein). This result indicates that the Chl-POX activity of treated fruit was significantly lower than the control fruit in the late period of storage (Fig. 1f). The MD activity range was 0.47–0.51 U mg^−1^ protein, and it was not significantly different for the two treatments from days 0 to 12. However, at the end of the storage period, E-beam-treated fruits showed significantly higher MD activity than the control (Fig. 1e). Thus, the present work demonstrates that the delay in chlorophyll degradation in the mango peel was caused by the E-beam treatment, which suppressed PPH and Chl-POX activities in the later stage of storage. However, E-beam treatment did not strongly affect Chlase and MD activities.

### Effect of E-beam irradiation on ethylene production and respiratory rate

**Fig. 3:**
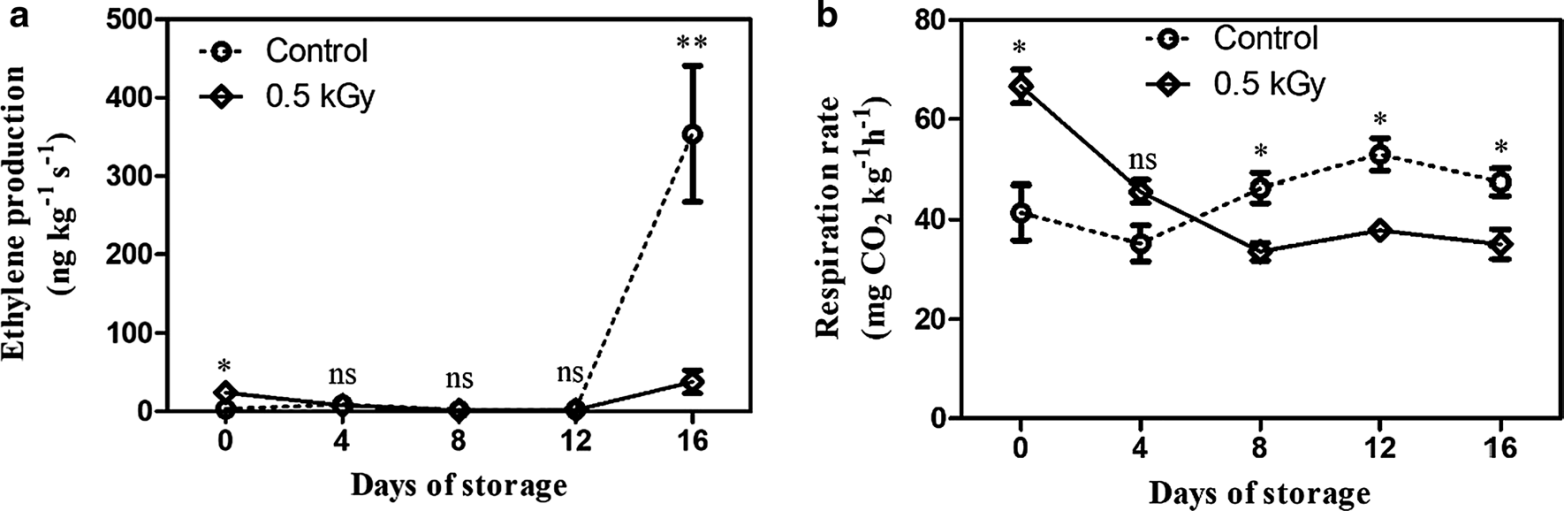
Ethylene production (**a**) and respiratory rate (**b**) of mango fruit after treatment with E-beam at a dose of 0 (control) and 0.5 kGy, and then stored at 13 °C for 16 days. The data are expressed as mean ± standard error. Asterisks (*) indicate significant differences between the two treatments during storage (t-test; *P < 0.05, **P < 0.01, *ns* not significant)

Notably, ethylene production increased immediately after the fruit was treated with E-beam (day 0), and it was significantly higher than that in the non-treated fruit. On days 4 to 12, ethylene production of the treated and non-treated fruit were not significantly different and remained stable within the range of 1.14–8.50 ng kg^−1^ s^−1^. Moreover, on day 16, the ethylene production in both fruit groups increased; the control fruit showed a rapid increase than the treated fruit (Fig. 3a). E-beam treatment increased the respiration rate by about 1.61-fold as compared to the non-treated fruits on day 0. After 8–16 days, the respiration rate of E-beam-treated fruit sharply decreased and became lower than that of the control (Fig. 3b).

The present results imply that the E-beam treatment triggered the ethylene production and respiration rate of mangoes in the early stage, which were later suppressed by the treatment.

### Effect of E-beam on ROS production

**Fig. 4:**
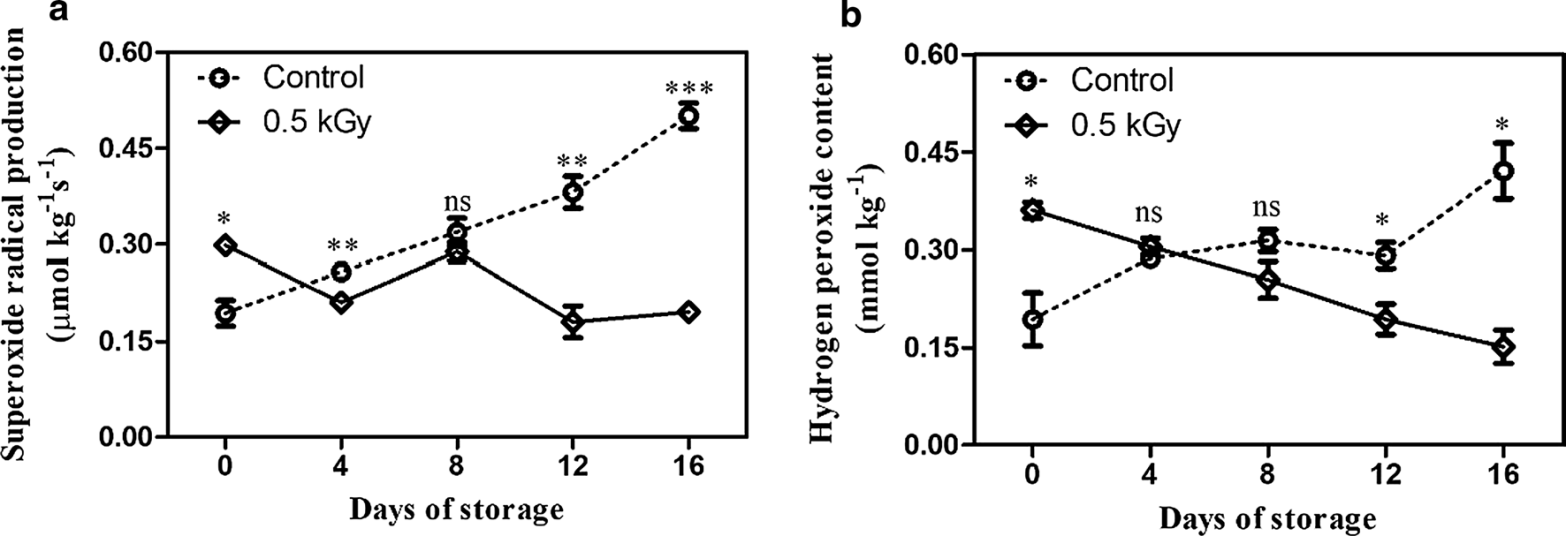
Superoxide radical (**a**) and hydrogen peroxide (**b**) contents of mango after treatment with E-beam at a dose of 0 (control) and 0.5 kGy, and then stored at 13 °C for 16 days. The data are expressed as mean ± standard error. Asterisks (*) indicate significant differences between the two treatments during storage (t-test; *P < 0.05, **P < 0.01, ***P < 0.001, *ns* not significant)

E-beam treatment induced the production of O^−**.**^_2_ immediately after implementation (0.30 μmol kg^−1^ s^−1^), which then declined during storage (0.19 μmol kg^−1^ s^−1^). In contrast, the O^−**.**^_2_ production in the control fruit increased throughout the period of storage (from 0.19 to 0.50 μmol kg^−1^ s^−1^). At the end of the storage period (day 16), O^−**.**^_2_ production in E-beam-treated fruit was approximately 2.6 times lower than that of the control fruit (Fig. 4a). E-beam also induced H_2_O_2_ content immediately after treatment: it was 0.36 mmol kg^−1^ on day 0, which was significantly higher than the control fruit (0.19 mmol kg^−1^). After this, a slight decline occurred until the end of the storage period (0.15 mmol kg^−1^). In contrast, the H_2_O_2_ content in untreated fruits tended to increase throughout the storage period. On day 16, the H_2_O_2_ content of the control fruit was 2.8-fold higher than that of the treated fruit (Fig. 4b). The results show that the E-beam treatment suppressed O^−**.**^_2_ production and H_2_O_2_ content in mango fruit during the storage period.

### Effect of E-beam on antioxidant capacity

**Fig. 5:**
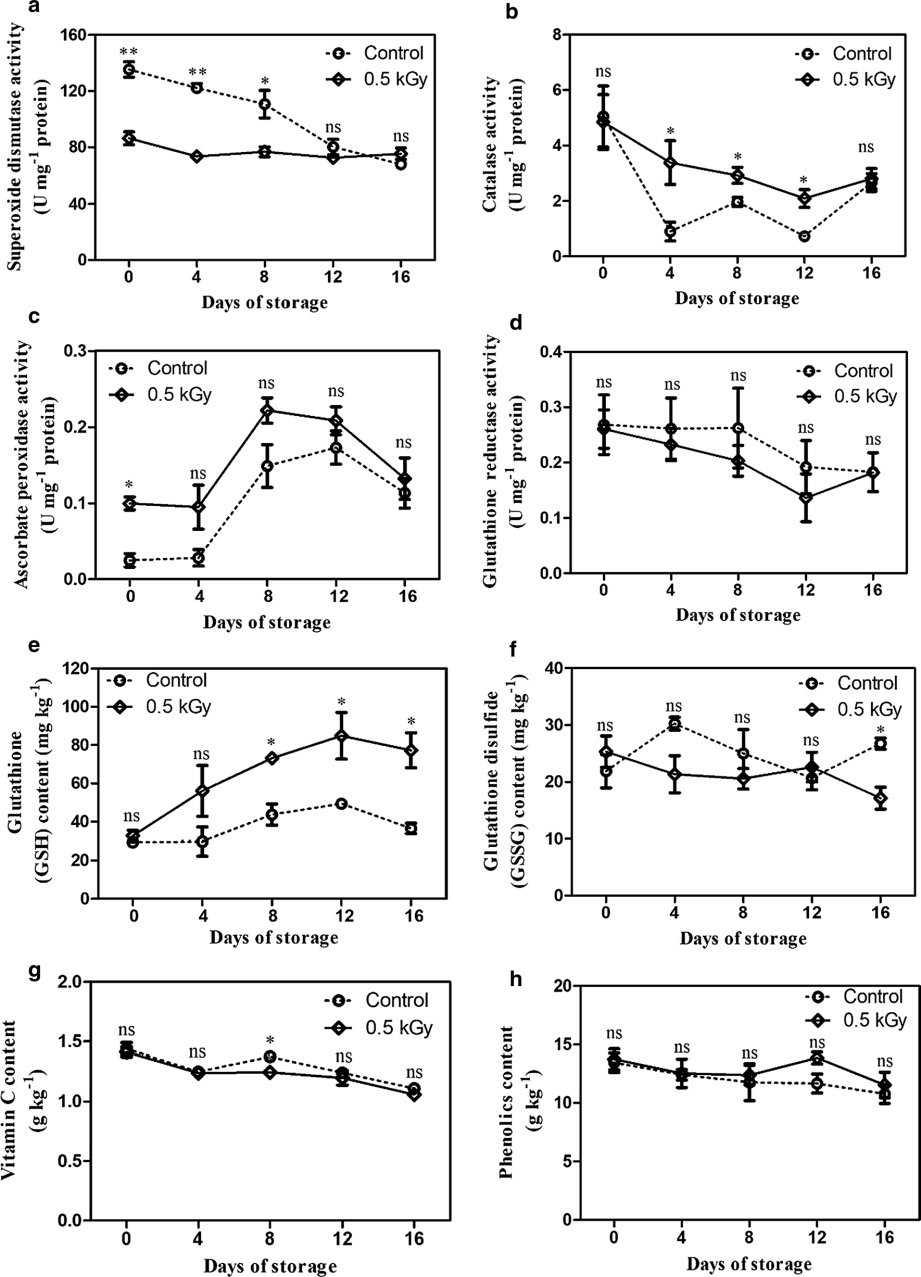
Superoxide dismutase (**a**), catalase (**b**), ascorbate peroxidase (**c**), glutathione reductase (**d**), glutathione content (**e**), glutathione disulfide (**f**), vitamin C content (**g**) and phenolic content of mangoes after treatment with E-beam at a dose of 0 (control) and 0.5 kGy, and then stored at 13 °C for 16 days. The data are expressed as mean ± standard error. Asterisks (*) indicate significant differences between the two treatments during storage (t-test; *P < 0.05, **P < 0.01, *ns* not significant)

The SOD activity in both the treated and control fruits tended to decline during storage. E-beam-treated fruits showed significant suppression in SOD activity from days 0 to 8 as compared to the control, while no significant differences were found on days 12 to 16 (Fig. 5a). CAT activity of both the treatments tended to decrease, and the E-beam-treated fruit had significantly higher CAT activity than the control fruit throughout the storage period except for the first and last few days (Fig. 5b). APX activities of both the treatment and control groups tended to increase during days 0–12 and peaked on day 12. They declined by the end of storage (day 16). E-beam-treated fruit had significantly higher APX activity on day 0 than the control fruit, followed by an insignificant difference between the E-beam-treated fruit and non-treated fruit until the end of storage (Fig. 5c). The GR activity tended to decrease in both the groups. E-beam-treated fruit had lower GR activity than untreated fruit but without any significant difference during the storage period (Fig. 5d).

The GSH content of the E-beam-treated fruit and control fruit increased from days 0 to 12 and then declined slightly by the end of the storage. The GSH content in the E-beam-treated fruit was significantly higher than that of the control fruit (Fig. 5e). The difference between the GSSG content of the E-beam-treated and control fruit was insignificant from day 0 to 12. On the last day of storage, the GSSG content of the E-beam-treated fruit was significantly lower than that of the control fruit (Fig. 5f). In the present study, vitamin C was found to slightly decrease in both the groups, but it was not a significant difference except on day 8 (Fig. 5g). The pattern of phenolic content was similar to the vitamin C content. The phenolic content of the treated fruit and control slightly decreased, and there were no significant differences (Fig. 5h).

## Discussion

The color of a mango is an important and valid criterion to assess its quality, and it plays a crucial role in consumer acceptability. After mangoes are harvested, several biochemical changes are involved in the ripening process, such as an increase in the respiratory rate, ethylene production, and fruit softening as well as pigment changes. Medlicott et al. [42] reported that the mango peel color changes from green to yellow while ripening, which is accompanied by a chlorophyll breakdown. The present research shows that E-beam induces chlorophyll-degrading enzyme activity, particularly that of Chl-POX, in the early stages (day 4), which then sharply decreases until the end of storage. The Chl-POX activity was significantly lower in the treated fruits than the non-treated fruits. In addition, E-beam irradiation was found to reduce the activity of PPH and significantly increase MD at the end of the storage. However, in this study, E-beam had no effect on Chlase activity, implying that E-beam irradiation may delay chlorophyll degradation via the suppression of Chl-POX and PPH activities at the end of storage. Previous researches have shown that ionizing gamma irradiation maintains chlorophyll content in quince fruit [18], tomatoes [19], plums [20], and pears [21]. This may be caused by the effect of irradiation on chlorophyll-degrading enzymes, as seen in this work. Ethylene is a well-known plant hormone that accelerates plant senescence [32]. In the present work, E-beam elicited ethylene production immediately after treatment, and the production was strongly suppressed at the end of storage, leading to a delay in chlorophyll degradation. Thus, low ethylene production in E-beam-treated fruit may cause a delay in chlorophyll degradation.

It is known that ROS is generally formed by the respiratory process of living cells. The plant’s mitochondrial respiratory electron transport chain generates O^−**.**^_2_ as a byproduct during energy metabolism in complex I and complex III [43, 44]. The O^−**.**^_2_ is converted to H_2_O_2_ by SOD in the chloroplasts, peroxisomes, and mitochondria [45, 46]. Afterward, H_2_O_2_ combines with the reduced transition metal ions such as Fe^2+^ or Cu^+^ to generate OH^−**.**^ via the Fenton and Haber–Weiss reaction [47]. Tahergorabi et al. [48] also demonstrated that ROS can generate in plants by water radiolysis under E-beam ionizing irradiation. Our experiment shows that E-beam treatment triggers the increase of H_2_O_2_ and O^−**.**^_2_ immediately after treatment (day 0), which then gradually decreases throughout storage. Therefore, an increase of H_2_O_2_ and O^−**.**^_2_ on day 0 may be caused by high respiratory and water radiolysis process. However, previous studies report that treatments of ROS (O^−**.**^_2_, H_2_O_2_, and hydroxy radical (OH^−**.**^)) in higher plants are a cause of chlorophyll degradation due to the chlorophyll phytyl chain getting oxidized to form isophytol [15, 16]. Therefore, high chlorophyll degradation in E-beam-treated fruit on day 4 can be assumed to have been caused by high H_2_O_2_ and O^−**.**^_2_ production, and the low chlorophyll degradation from day 8 until the end of storage is related with the low content of H_2_O_2_ and O^−**.**^_2_.

The antioxidant system is known to play an important role in delaying chlorophyll degradation and plant senescence [49–51]. E-beam irradiation also affects the antioxidant capacity that participates in scavenging ROS in plants. The present study shows that E-beam irradiation suppresses SOD activity. Similarly, results of previous studies show that ionizing beta-irradiation or gamma irradiation retards SOD activity in mandarins [52], apricots [53], pepper [54], and *Zizania latifolia* [55]. The activities of CAT and APX in E-beam-treated fruit tended to increase during storage as compared to the control fruits in the present study. These results are in agreement with those of Zhang et al. [52], Duan et al. [56], Hong et al. [57], and El-Beltagi et al. [58], who respectively reported that ionizing irradiation triggers an increase in CAT and APX activities in mandarin, wheat, and rosemary.

Vitamin C is an antioxidant compound that preserves the quality and phytonutrient of a fruit, whereas GSH plays an important role in detoxification, antioxidant defense, thiol status maintenance, and cell proliferation modulation [59]. Both vitamin C and GSH are associated with the ascorbate–glutathione cycle, which plays a crucial role in protecting plant cells in response to stress [60]. Our results show that the vitamin C content decrease in both the E-beam-treated and non-treated fruits without any significant differences. In contrast, the GSH content increase in a similar pattern in both the groups from days 0 to 12, with a slight decrease toward the end of the storage period. Similar results were demonstrated by Kim and Yook [61] and Maraei and Elsawy [62], indicating that vitamin C content in harvested kiwis and strawberries does not get affected by gamma irradiation. Further, Erkan et al. [63] reported that UV irradiation induces an increase in GSH in strawberries. GR is an ubiquitous NADPH-dependent enzyme that converts GSSG to GSH in the ascorbate–glutathione cycle [60]. The present results indicate that E-beam treatment does not affect GR activity during storage. Moreover, the present research shows that E-beam treatment does not affect phenolic compounds. These results suggest that E-beam has the potential to apply on mango fruits and other fruits for delaying ripening and senescence such as our recent research in lime fruit [64]. It can be used as the optional treatment of ionizing gamma ray. Because the limits of gamma irradiation are high cost, difficult to operation as compared with E-beam irradiation. Since the radioactive isotopes (^60^Co, harmful) is required to generate gamma ray where E-beam uses the electricity as the energy source to generate E-beam [65] which it is much safer than using radioactive nuclides. However, the basic research information of E-beam treatment for mango industry still limits. Its application for controlling physiochemical changes, insect infestation and postharvest diseases are required in further study.

## Supplementary Information

**Additional file 1: Figure S1.** E-beam irradiator that was used in this experiment. **Figure S2.** The illustration of dosimeters set up for dose mapping of mangoes in an E-beam process. **Figure S3.** Video shows the loading process of mango for E-beam irradiation. **Figure S4.** Alanine pellet dosimeters (A and B) and electron spin resonance spectroscopy (E-scan™) for alanine dosimeter reader (C) that were used in this experiment.

## Data Availability

The datasets used and/or analysed during the current study are available from the corresponding author on reasonable request.
