# Supplementary material for: Impact of electron beam irradiation on the chlorophyll degradation and antioxidant capacity of mango fruit
Source: Appl Biol Chem. 2021 Feb 3;64(1):19. doi: 10.1186/s13765-021-00592-8 (PMC7854327; doi:10.1186/s13765-021-00592-8)
Supplement: Supplementary file 1 — Additional file 1: Figure S1. E-beam irradiator that was used in this experiment. Figure S2. The illustration of dosimeters set up for dose mapping of mangoes in an E-beam process. Figure S3. Video shows the loading process of mango for E-beam irradiation. Figure S4. Alanine pellet dosimeters (A and B) and electron spin resonance spectroscopy (E-scan™) for alanine dosimeter reader (C) that were used in this experiment. [file 13765_2021_592_MOESM1_ESM.docx]

| **Additional File 1**  **Impact of Electron Beam Irradiation on the Chlorophyll Degradation and Antioxidant Capacity of Mango Fruit**  Truc Trung Nguyen; Apiradee Uthairatanakij; Varit Srilaong, Natta Laohakunjit,  Masaya Kato, and Pongphen Jitareerat |
| --- |

| 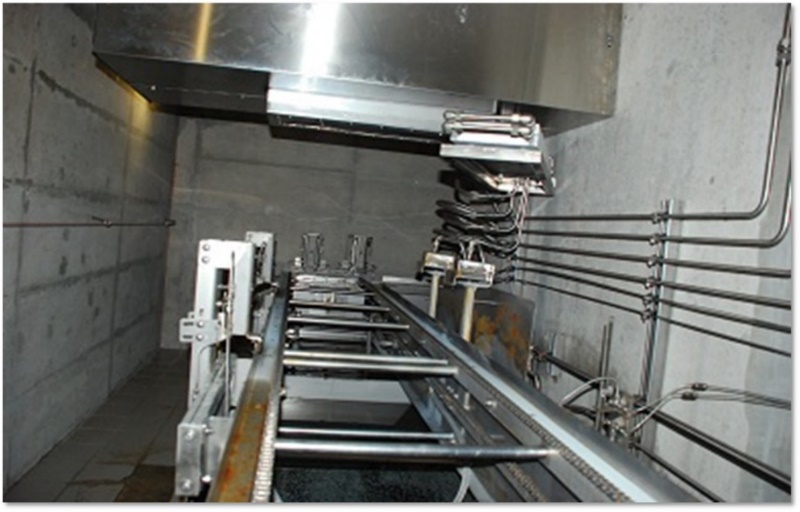 | 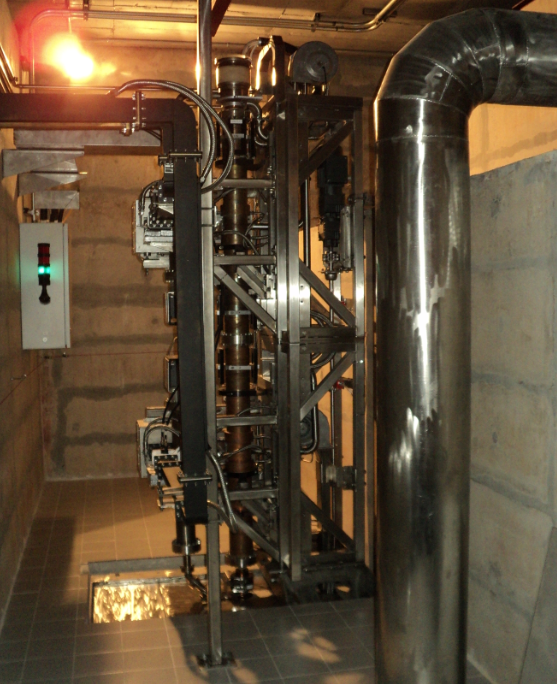 |
| --- | --- |
| **Figure S1.** E-beam irradiator that was used in this experiment. | |
| 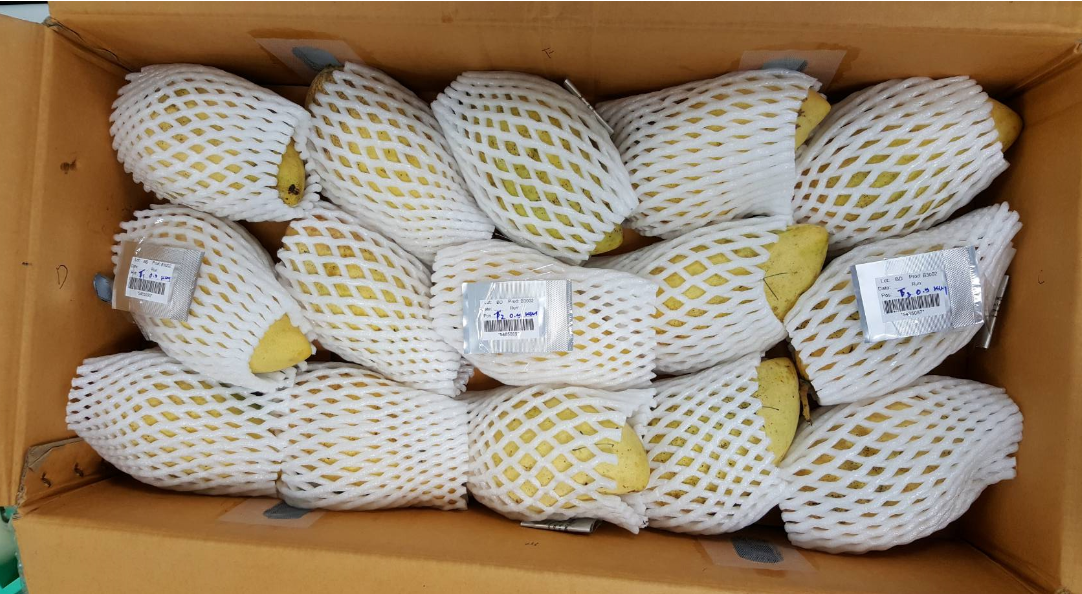 | |
| **Figure S2.** The illustration of dosimeters set up for dose mapping of mangoes in an E-beam process. | |

**Figure S3.** Video shows the loading process of mango for E-beam irradiation.

| 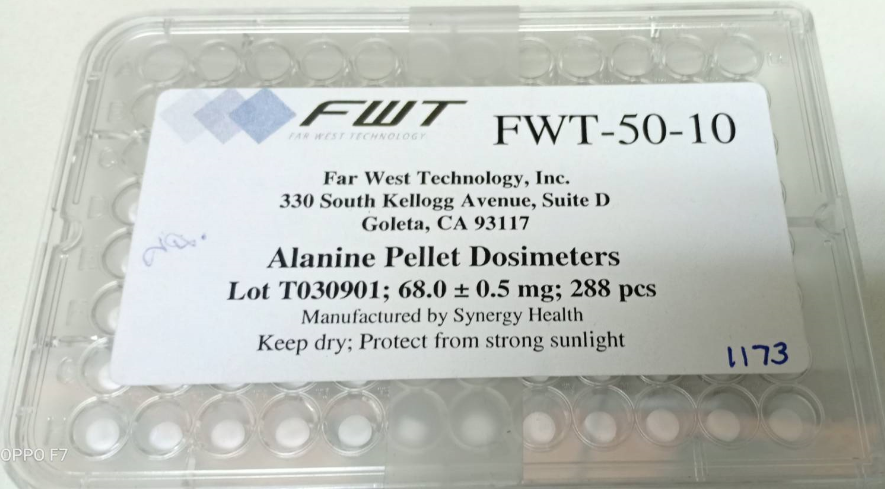  (A) | 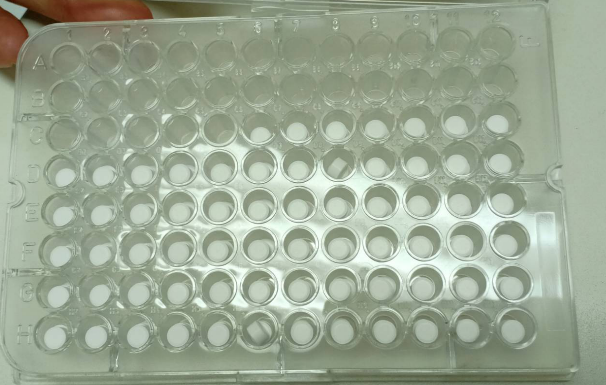  (B) |
| --- | --- |
| 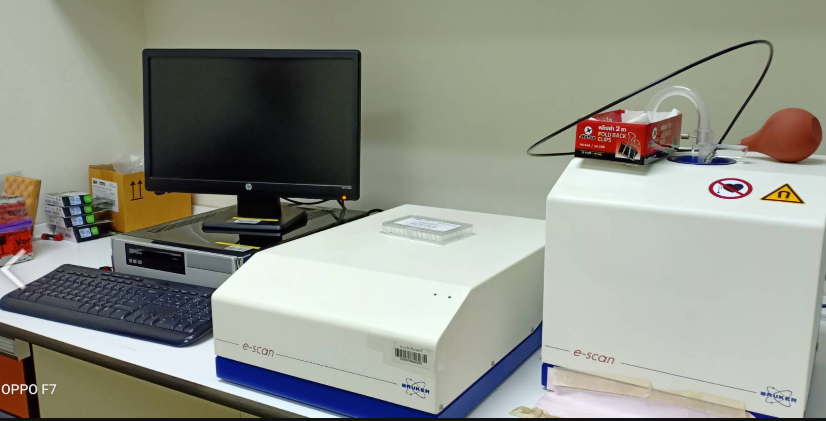  (C) | |
| **Figure S4.** Alanine pellet dosimeters (A and B) and electron spin resonance spectroscopy (E-scan™) for alanine dosimeter reader (C) that were used in this experiment. | |
